# The probiotic *Bacillus subtilis* BS50 decreases gastrointestinal symptoms in healthy adults: a randomized, double-blind, placebo-controlled trial

**DOI:** 10.1080/19490976.2022.2122668

**Published:** 2022-10-21

**Authors:** Sean M. Garvey, Eunice Mah, Traci M. Blonquist, Valerie N. Kaden, Jessica L. Spears

**Affiliations:** a BIO-CAT Microbials, LLC, Shakopee, MN, USA; b BIO-CAT, Inc., Troy, VA, USA; c Biofortis Research, Inc., Addison, IL, USA

**Keywords:** Bacillus subtilis, probiotic, gastrointestinal, digestion, burping, bloating

## Abstract

Durable spore-forming probiotics are increasingly formulated into foods, beverages, and dietary supplements. To help meet this demand, the safety and efficacy of daily supplementation of *Bacillus subtilis* BS50 for 6 weeks was investigated in a randomized, double-blind, placebo-controlled, parallel clinical trial of 76 healthy adults. Before and during supplementation, gastrointestinal symptoms were recorded daily using a multi-symptom questionnaire. Clinical chemistry, hematology, plasma lipids, and intestinal permeability and inflammation markers were measured at baseline and end of study. Compared to placebo, 2 × 10^9^ colony-forming units (CFU) BS50 per day increased the proportion of participants showing improvement from baseline to week 6 in the composite score for bloating, burping, and flatulence (47.4% vs. 22.2%), whereby the odds of detecting an improvement were higher with BS50 (OR [95% CI]: 3.2 [1.1, 8.7], p = .024). Analyses of individual gastrointestinal symptoms indicate that BS50 increased the proportion of participants showing an improvement at week 6 compared to placebo for burping (44.7% vs. 22.2%, p = .041) and bloating (31.6% vs. 13.9%, p = .071), without affecting other symptoms. There were no clinically meaningful changes in clinical chemistry, hematology, plasma lipids and intestinal permeability and other inflammation markers. In conclusion, the results suggest that dietary supplementation of 2 × 10^9^ CFU *Bacillus subtilis* BS50 per day is a well-tolerated and safe strategy to alleviate gas-related gastrointestinal symptoms in healthy adults.

**Abbreviations:**

AE adverse event; BHD bowel habits diary; BMI body mass index; BSS Bristol Stool Scale; CFU colony-forming unit; CRP C-reactive protein; FGID functional gastrointestinal disorder; GI gastrointestinal; GITQ Gastrointestinal Tolerance Questionnaire; GLP-1 glucagon-like peptide 1; GSRS Gastrointestinal Symptom Rating Scale; HDL-C high-density lipoprotein-cholesterol; IBS irritable bowel syndrome; IL-10 interleukin-10; ITT intent-to-treat; LBP lipopolysaccharide binding protein; LDL-C low-density lipoprotein-cholesterol; PP per protocol; PYY peptide YY; TG triglyceride; total-C total cholesterol

## Introduction

Gastrointestinal (GI) symptoms such as abdominal bloating are commonly reported in otherwise healthy adults, most often in females, and are more severe in those with digestive diseases and functional GI disorders (FGIDs).^1,2^ In the 2015 National GI Survey of 71,812 community-dwelling adults in the United States inclusive of comorbid conditions, 61% of respondents reported having at least one of eight specific GI symptoms over the prior week.^3^ The top three reported symptoms were heartburn/reflux (31%), abdominal pain (25%), and bloat/gas (21%). Consistent with these results, in a 1997 survey of 2,510 adults, 41% reported having at least one symptom of abdominal pain or discomfort (22%), bloating or distension (16%), or loose stools or diarrhea (27%) over the prior month.^4^ Among those respondents with bloating or distension, more than 50% reported a reduction in usual daily activities and 43% took medications such as antacids and anti-gas medications.^4^ A separate 2003 bloating-specific survey of 2,259 adults suggested the prevalence of abdominal bloating to be 27% overall and 19% when adjusted for the age and sex of the 2000 United States population.^5^ Abdominal bloating and symptoms related to gas – flatulence and burping – thus represent a significant burden that impacts quality of life in the general population.^4^ Furthermore, there remains a paucity of interventional trials aiming to reduce gas-related symptoms such as abdominal bloating and flatulence in healthy participants without FGID.

Abdominal bloating is defined as “the subjective sensation of gassiness, trapped gas, or a feeling of pressure or being distended without obvious visible distension”.^6^ Bloating can also occur with objective physical distension of the abdomen. The etiology for bloating and distension is complex and multifactorial. Triggers may include disturbances to digestive enzyme output and activity, intestinal transit and motility, composition of the intestinal microbiota, intestinal gas production, immune function, visceral hypersensitivity, and central nervous system processing.^7,8^ Bloating has also been described as primarily a sensory phenomenon associated with a lower pain threshold or increased biological sensitivity, as evidenced by computed tomography imaging showing that luminal gas increases in only 25% of FGID patients during a bout of abdominal distension or following consumption of a “high-flatulence” diet.^9^ These results point to a potential role of the gut-brain axis in perception of bloating severity, and suggest that the intestinal microbiota or gut sensory neuropod cell signaling modulation could impact hypersensitivity and bloating. Related to gut-brain cross-talk, belching can be the consequence of aerophagia, or swallowing air, which itself is influenced by anxiety, depression, and hypervigilance.^10^

Given the side effects of common over-the-counter medications to reduce bloating and distension, dietary supplements have been considered as alternatives and complements. It is estimated that 57 to 80% of adults in the United States consume dietary supplements, ranging from category-leading multivitamins to benefit-specific products to help support immunity and digestive health.^11,12^ Many digestive health products contain probiotics that complement endogenous, beneficial gut microbes and digestive enzymes. Probiotics are live microorganisms that, when administered in adequate amounts, confer a health benefit on the host.^13^ Examples of probiotic health benefits include the support of digestive health, GI health, intestinal microbiota balance, immunity, and mood.^14,15^ Species of the *Bacillaceae* family are particularly suited for probiotic applications because they can be manufactured as highly durable endospores, or spores, with thick proteinaceous coats. Spores can persist without refrigeration, survive desiccation and heat exposure, and resist the acidic and high bile salt conditions that occur throughout the mammalian GI tract.^16,17^ Several *Bacillaceae* strains have been demonstrated to be safe for human consumption. For example, five *Bacillus subtilis* strains and six *Weizmannia coagulans* (formerly *B. coagulans*) strains are “generally regarded as safe” (GRAS) for use in food without objection from the United States Food and Drug Administration (FDA).^18–28^ Additionally, the European Food Safety Authority (EFSA) has included *B. subtilis* on the qualified presumption of safety (QPS) list of biological agents, allowing their use in food.^29^ *B. subtilis* and *W. coagulans* strains have also been clinically shown to support digestion and GI health in participants with symptoms of FGID, including inflammatory bowel syndrome (IBS) and dyspepsia,^30–38^ as well as healthy participants.^39–41^ The GI-directed probiotic activity of *Bacillaceae* species is likely mediated by the secretion of digestive enzymes (e.g., protease, amylase, xylanase) and antimicrobial compounds (e.g., bacteriocins, fengycins, surfactins) that contribute to digestion, intestinal microbiota balance, and immunity.^42,43^

Consistent with the prevalence of GI symptoms among the general population, there remains a need to study the effects of probiotics on GI symptoms in healthy participants without FGID such as IBS. Following a multi-year probiotic strain screening and profiling program of a library of over 1,000 soil-derived *Bacillaceae* species, *B. subtilis* strain BS50 (BS50) was advanced for clinical study in healthy adults. The safety of BS50 has been demonstrated utilizing published scientific procedures.^44,45^ *In silico* and *in vitro* analyses of BS50 indicate that the BS50 genome does not encode any known *Bacillus* toxins.^44^ The BS50 genome contains several gene clusters involved in the biosynthesis of secondary metabolites, many of which are antimicrobial metabolites such as fengycins that may confer health benefits related to intestinal microbiota balance. Additionally, BS50 cell lysates do not negatively impact cultured human Caco-2 intestinal epithelial cell viability or monolayer permeability.^44^ BS50 spore preparations show robust heat resistance and pH tolerance (unpublished data), which predict strain survival across a wide range of pH across the human gastric and intestinal compartments. BS50 also secretes a suite of extracellular enzymes in its vegetative state, which is likely to support postprandial digestion, nutrient absorption, and GI tolerance.

We conducted a randomized, double-blind, placebo-controlled trial to study the safety and effect of BS50 on three primary GI symptoms – abdominal bloating, burping, and flatulence – in healthy adults. The effects of BS50 on bowel habits, stool consistency, circulating intestinal permeability markers, circulating inflammatory markers, lipid profile, and sleep quality were also investigated. We hypothesized that 2 × 10^9^ CFU BS50 supplementation once daily with the largest meal of the day for 6 weeks would increase the proportion of participants showing an improvement in the composite score of abdominal bloating, burping, and flatulence.

## Materials and methods

### Study design

The study was conducted in accordance with the Declaration of Helsinki, the United States FDA Code of Federal Regulations, Title 21 and the International Code of Harmonization (E6) Good Clinical Practice Guidelines after approval of Protocol No. BIO-2112 and informed consent documents on 12 July 2021 by the Institutional Review Board at Advarra, Inc. (Columbia, MD, USA). Signed informed consent and authorization for use of protected health information were provided by the participants before implementing any protocol-specific procedures. The study was prospectively registered at ClinicalTrials.gov (NCT05004454) and conducted between July and November 2021 at a single clinical research site (Biofortis Research; Addison, IL, USA).

This was a randomized, double-blind, placebo-controlled, parallel-group study with two treatment arms. Participants (n = 76) consumed one capsule of BS50 (2 × 10^9^ CFU/capsule) or a matching maltodextrin placebo capsule daily with their largest meal for 6 weeks. The week before supplementation and throughout the 6-week supplementation period, GI symptoms and bowel habits were recorded using a Gastrointestinal Tolerance Questionnaire (GITQ) and Bowel Habits Diary (BHD), respectively. Additionally, sleep quality and the presence and duration of any respiratory infection were assessed with a Sleep Quality and Respiratory Infection Questionnaire. Before and at the end of the 6-week supplementation period, plasma GI permeability markers (zonulin, occludin, and lipopolysaccharide-binding protein [LBP]), plasma inflammatory markers (C-reactive protein [CRP], interleukin-8 [IL-8], IL-6, IL-10, interferon-gamma [IFN-γ], and tumor necrosis factor-alpha [TNF-α]), and fasting plasma lipid profile (triglyceride [TG], total cholesterol [total-C], LDL-C, and HDL-C) were assessed.

### Study participants and screening

Participants were healthy 30–65 year-olds with a BMI of 18.0–31.9 kg/m^2^ who consumed a typical American diet and had at least minimal complaints of abdominal bloating, burping, or flatulence defined as having a combined score of 3 or more for abdominal bloating, burping, and flatulence as assessed using the GITQ during the baseline week before the start of supplementation (i.e., week −1). Only one of the 101 screened participants failed to meet this inclusion criterion. Additionally, participants were nonsmokers, had no history of major illness, no clinically important GI conditions, were not pregnant, and were using contraception to prevent pregnancy during the study (females only), with no recent antibiotic use (< 3 months within screening Visit 1), no recent use of medications or products (e.g., probiotic supplements) known to influence GI function, and no known allergies to any of the study product ingredients. At screening (Visit 1, day −7), participants completed a medical history questionnaire in addition to the assessment of height, weight, BMI, vital signs, last menses (females only), current medication/supplement use, and review of inclusion/exclusion criteria to determine eligibility. Females under the age of 60 years completed a urine pregnancy test. In addition, fasting blood samples were collected at Visit 1 for analysis of clinical chemistry and hematology. One week later at Visit 2 (day –1), eligible participants were randomized (1:1) to one of the study groups, based on a statistician-generated allocation sequence using a permuted blocks algorithm in SAS PROC PLAN, stratified by sex and BMI. The sequence was uploaded onto the electronic case report form platform (Medrio Inc.; San Francisco, CA, USA). Participants were asked to maintain habitual exercise, diet, and medication/supplementation use during the study.

### Study products

*Bacillus subtilis* BS50 (also known as *B. subtilis* BS8-74) is a gram-positive, spore-forming bacterium that was isolated at BIO-CAT Microbials, LLC (Shakopee, MN, USA) from soil collected from Gallatin County, Montana, USA on 4 July 2015.^44^ BS50 has been deposited in the American Type Culture Collection with Accession No. PTA-127287. The BS50 spore powder was manufactured by BIO-CAT Microbials (Shakopee, MN, USA) under food-safe cGMP conditions. Both the probiotic and placebo study products were manufactured into capsules by Vitaquest International LLC (West Caldwell, NJ, USA), an FSSC 22000 facility. Each probiotic capsule contained 2 × 10^9^ CFU of *B. subtilis* BS50 with identity-preserved maltodextrin extracted from waxy maize as the excipient. This dose was selected based on published clinical trials demonstrating that doses as little as 1 × 10^9^ CFU/day and up to 5 × 10^9^ CFU/day of *Bacillaceae* strain probiotics yielded clinically meaningful results.^30–35,37–41^ The placebo capsule only contained identity-preserved maltodextrin. Both products were manufactured into opaque, white, size 1 capsules made from pharmaceutical cellulose ethers from vegetable sources (hydroxypropyl methylcellulose) and titanium dioxide E171 and were identical in appearance. Study products were provided to participants in bottles containing 50 capsules each.

Every day for 6 weeks, participants were instructed to consume the study product once a day (1 capsule/day) with their meal which was typically the largest of the day. For example, if lunch was typically the largest meal of the day for an individual participant, that participant was instructed to consume the study product with every lunch for 6 weeks starting at week 1. If a participant failed to consume the study product at the appropriate meal (due to not remembering, etc.), the participant was instructed to consume the product with the next meal (e.g., dinner, if missed at lunch) or with a snack (e.g., evening snack, if missed at dinner). Participants were advised not to consume more than 1 capsule/day. Compliance was assessed at the end of 6 weeks by the counting of returned unused products. Participants were also instructed to complete a daily study product log, a procedure used to increase compliance. Satisfactory compliance was defined as product intake between 80% and 120%.

### Study questionnaires

#### Study product log

A daily study product log queried compliance with study product intake. Participants documented in the paper log if they had consumed the study products and the time of consumption.

#### Gastrointestinal Tolerance Questionnaire (GITQ)

The GITQ contained a series of questions regarding the presence and severity of eight GI symptoms occurring during the past 24 hours.^46^ These GI symptoms included gas/flatulence, nausea, vomiting, abdominal cramping, abdominal distention/bloating, borborygmus/stomach rumbling, burping, and reflux (heartburn). Severity was ranked on a 4-point scale ranging from none (score: 0) to severe (score: 3). Participants were required to complete a paper GITQ daily for 7 days before the start of supplementation (i.e., week −1) to determine eligibility. During these 7 days, participants must have had at least one occurrence of abdominal bloating, burping, or flatulence and a combined weekly total symptom score for abdominal bloating, burping, and flatulence of ≥ 3 (e.g., 3 days with a mild severity across the three symptoms, 1 day of mild severity and 1 day of moderate severity across the three symptoms, or 1 day of severe severity across the three symptoms). To capture changes in GI symptoms during the supplementation period, participants were instructed to complete the GITQ electronically daily (Medrio Inc.; San Francisco, CA, USA) from week 1 through week 6.

#### Bowel habits diary (BHD)

The BHD was used to collect information on stool frequency and consistency, straining and discomfort during bowel movements, and any sensation of incomplete evacuation. Participants were required to complete the BHD during the 7 days leading up to the start of supplementation (i.e., week −1) for baseline information and daily during the 6-week supplementation period (i.e., week 1 through week 6). The degree of these bowel-related symptoms was ranked on a 4-point scale ranging from none (score: 1) to severe (score: 4). Stool consistency was rated from 1 to 7 (solid to liquid) according to the Bristol Stool Scale (BSS).^47^ Participants were also required to record all bowel movement occurrences during baseline and supplementation periods. Responses for baseline were manually entered into the database while responses during the supplementation period were recorded digitally (Medrio Inc.; San Francisco, CA).

#### Sleep quality and respiratory infection questionnaire

Participants completed a brief questionnaire on sleep quality and the presence and duration of any cold/flu episode weekly. Each week, participants ranked their sleep quality over the past week from 0 to 10 (terrible to excellent), as described in the Single-Item Sleep Quality Scale.^48^ Participants were also required to recall if they had any episode of cold/flu/respiratory infection over the past week and if present, the number of days symptoms were experienced. A paper-based questionnaire was completed in the clinic for the 7 days leading up to the start of supplementation (i.e., week −1). For the remaining study duration, the questionnaire was completed at home and responses were recorded digitally (Medrio Inc.; San Francisco, CA, USA) from week 1 through week 6.

#### In-clinic procedures

For testing visits at the end of week −1 (Visit 2, day –1) and week 6 (Visit 3, day 42), participants reported to the clinic after an overnight fast (at least 10 hours) and having avoided exercise for 24 hours. On arrival, vital signs (seated, resting blood pressure, and heart rate) were measured using an automated device, body weight, last menses query (females only), and current medication/supplement use were assessed, and compliance with study instructions and eligibility criteria was reviewed. Fasting blood samples were collected and processed for the following analyses: intestinal permeability markers (zonulin, occludin, and LBP), inflammatory markers (CRP, IL-8, IL-6, IL-10, IFN-γ, and TNF-α), and lipid profiling (TG, total-C, HDL-C, LDL-C). Participants were queried about adverse events (AE) that occurred since their last study visit at the beginning of each visit and graded as mild (awareness of symptoms but easily tolerated), moderate (discomfort enough to interfere with but not prevent daily activity), or severe (unable to perform usual activity) by the study physician. Additionally, the likelihood that an AE was related to the study product was classified as not related, unlikely, possibly, probably, or definitely. At the end of week −1 (Visit 2), participants were provided with a supply of study products to take home. At week 6, participants returned to the clinic for assessment of vitals and fasting blood sample collection as described above as well as for clinical chemistry and hematology.

#### Biological sample analysis

Clinical chemistry, hematology, and lipid profile analysis were performed by Elmhurst Memorial Reference Laboratory (Elmhurst, IL, USA). Clinical chemistry included albumin, aspartate aminotransferase, alanine aminotransferase, alkaline phosphatase, total bilirubin, calcium, chloride, creatinine, blood urea nitrogen, potassium, sodium, total protein, carbon dioxide, osmolality, and glucose. Hematology included white blood cell count, red blood cell count, hemoglobin concentration, hematocrit (as volume percent), mean cell volume, mean cell hemoglobin concentration, neutrophils, lymphocytes, monocytes, eosinophils, basophils, and platelet count. Serum chemistry, hematology, TG, total-C, and HDL-C were assessed using the Dimension Vista® System (Siemens Healthcare; Erlangen, Germany). LDL-C was calculated according to the Friedewald equation.^49^ Normal ranges for all values were provided by the testing lab and accounted for each participant’s age and gender.

Plasma IL-8, IL-6, IL-10, IFN-γ, and TNF-α were analyzed in duplicate using the U-PLEX Biomarker Group 1 Human assay kit (catalog no. K15067L-1, Meso Scale Diagnostics, LLC; Rockville, MD, USA), and plasma CRP was analyzed in duplicate using the V-PLEX Human CRP kit (catalog no. K151STD-1, Meso Scale Diagnostics, LLC). All analyses were performed per the manufacturer’s recommendation by PBL Assay Science (Piscataway, NJ, USA). Plasma zonulin (catalog no. EKC 36091, Biomatik USA, LLC; Wilmington, DE, USA), occludin (catalog no. NBP2-80305, Novus Biologicals, LLC; Centennial, CO, USA), and LBP (catalog no. DY870-05, R&D Systems, Inc.; Minneapolis, MN, USA) were analyzed in duplicate by ELISA by the Institute for Food Safety and Health (Illinois Institute of Technology; Chicago, IL, USA) according to manufacturer’s instructions.

### Statistical methods

A sample of 64 participants was expected to provide 80% power (alpha = .05, two-tailed) to detect a difference between groups of 30% in the proportion of participants with improvements in the 3-item composite score of flatulence, bloating, and burping (primary outcome variable). A sample of 76 participants was randomized to allow for attrition and noncompliance.

All statistical analyses were conducted using SAS for Windows (version 9.4; Cary, NC, USA). Tests of significance were two-sided and performed at the .05 significance level. The statisticians performing the analysis and scientific investigators remained blinded until after the completion of all statistical analyses. Primary analysis was completed for the intent-to-treat (ITT) population which included all participants who were randomized into the study. In addition, analyses were conducted on compliant participants who completed the study (per protocol [PP] population). All analyses of study samples were identified prior to locking the database. There were few qualitative differences in the results between the ITT and PP populations, and thus, the results presented herein are from the ITT population unless otherwise stated.

The proportion of participants that had an improvement in the 7-day, 3-item total composite score of flatulence, bloating, and burping were compared between groups with the chi-square test. An improvement was defined if the end of study 3-item composite score decreased by at least 2 points as compared to baseline where any of the 3 individual items did not increase by 1 or more points. Additionally, the odds of observing an improvement in the 7-day, 3-item composite score were then modeled with logistic regression and adjusted for sex and BMI category. The proportion of participants with an improvement in each GI symptom, defined as a score change ≤ −1, as well as the proportion of participants who reported good to excellent sleep in a given week, was compared between groups with a chi-square test; if the cell count was < 5, a Fisher’s exact test was used.

The weekly average stool consistency and the weekly number of bowel movements were analyzed with a repeated-measures model and generalized linear mixed model following a Poisson distribution with a log link, respectively. The model contained fixed effect terms for product, week, and product by week interaction, sex, and BMI category. The change from baseline for symptoms reported on the bowel habits diary was compared between groups at each week with the Wilcoxon rank-sum test.

Blood lipids, markers for intestinal permeability, and inflammation markers were compared between products with the analysis of covariance (ANCOVA) approach. The change from baseline was used as the outcome and adjusted for baseline, product, sex, and BMI group. For the inflammatory markers, model assumptions were violated and the rank transformation was used. As a sensitivity analysis, the Wilcoxon rank-sum test and the stratified Wilcoxon test were also used to evaluate differences between BS50 and placebo at the end of baseline week −1 (day –1) and end of week 6 (day 42), and the change from day –1 to day 42.

The within-group paired change from baseline (day −7) to week 6 (day 42) for clinical chemistry and hematology were compared with the Wilcoxon sign rank test. A false discovery rate (FDR; q- value) adjustment using the Benjamini-Hochberg procedure was used to control for multiple testing. If the within-group paired change was significant (q < .05), then the within-subject change in safety clinical chemistry and hematology was compared between products with the Wilcoxon rank-sum test.

## Results

### Disposition, participant characteristics, and compliance

**Figure 1.**
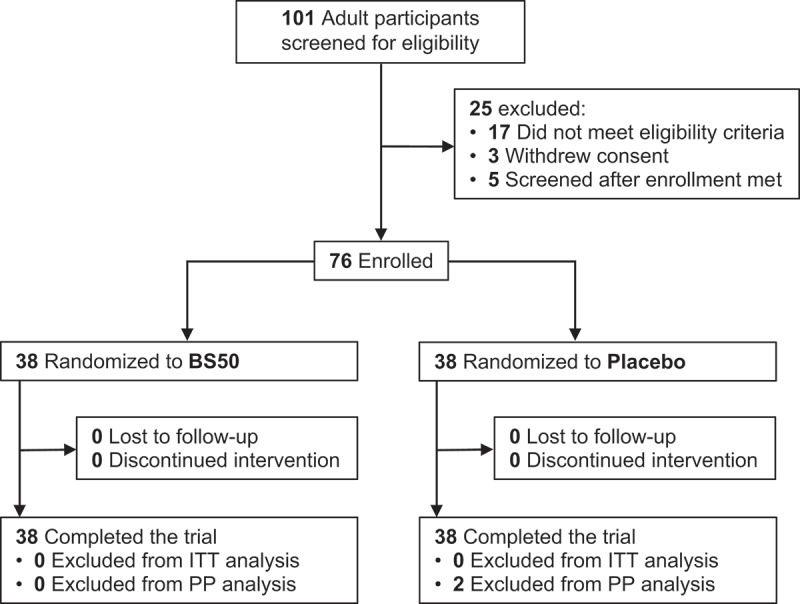
Participant enrollment and treatment assignment to *B. subtilis* BS50 probiotic (2 × 10^9^ CFU/day) or placebo. ITT, intent-to-treat; PP, per protocol.

A total of 76 participants were randomized to the BS50 or placebo arm, and all completed the study protocol in its entirety (Figure 1). The ITT population (n = 76) consisted of all randomized participants and the PP population consisted of 74 participants whereby two participants were removed due to concomitant medication use. Participant anthropometric characteristics at baseline for the ITT population are shown in Table 1. The compliance for BS50 was (mean ± SD) 100.9 ± 5.2% and the compliance for placebo was 101.0 ± 6.2%.

**Table 1.** Baseline anthropometric characteristics of randomized participants in the BS50 and placebo groups.

Characteristics	BS50 (n = 38)	Placebo (n = 38)
Female	20 (52.6%)	22 (57.9%)
Male	18 (47.4%)	16 (42.1%)
Age (years)	50.4 (10.0)	50.5 (8.8)
BMI (kg/m^2^)	25.8 (3.8)	25.8 (3.6)
Systolic blood pressure (mm Hg)	117.7 (11.8)	119.4 (12.3)
Diastolic blood pressure (mm Hg)	75.8 (8.3)	75.4 (8.5)
Fasting glucose (mg/dL)	94.0 (9.6)	92.3 (7.3)

Values are count (%) or mean (standard deviation)

BMI, body mass index

### Gastrointestinal symptoms

**Figure 2.**
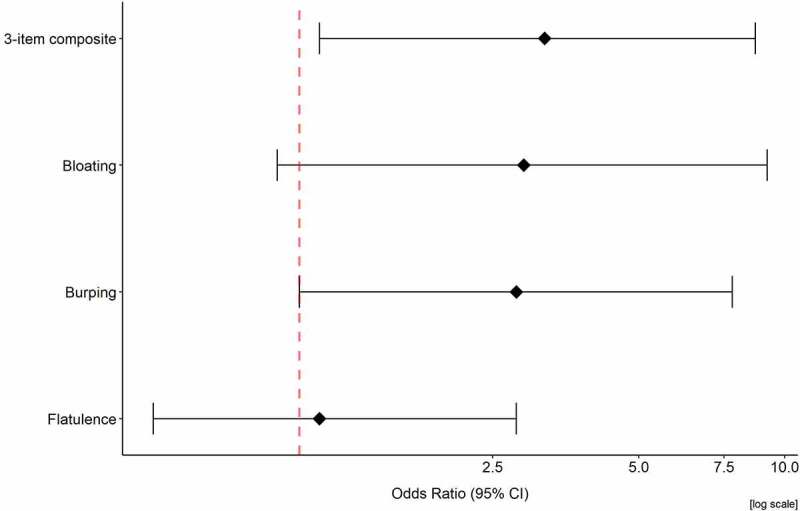
Odds ratio for the improvement in the 3-item composite, bloating, burping, and flatulence scores from baseline to week 6 for BS50 compared to placebo.

A significant difference (47.4% vs. 22.2%) was detected in the proportion of participants with an improvement of 2 or more points in the 7-day, 3-item composite score (i.e., composite score for flatulence, bloating, and burping) between week −1 and week 6, whereby the odds of detecting an improvement were higher (p = .024, chi-square; p = .026, logistic regression adjusted for sex and BMI group) following BS50 supplementation compared to placebo (Figure 2). Compared to placebo, the proportion of participants with an improvement of 1 or more points was greater following BS50 for burping (44.7% vs. 22.2%; p = .041) or bloating (31.6% vs. 13.9%; p = .071) but there were no significant differences between groups for flatulence (47.4% vs. 44.4%; p = .80). There were no significant differences in the proportion of participants with or without improvement for the remaining GI symptoms (Table 2). When evaluating the trend over time of the 7-day, 3-item composite score, no significant differences were detected at the individual time points (Supplemental Table S1). Although not significant, the point estimate and confidence interval favored BS50 at week 6, indicating a possible trend of a greater decrease in the mean change score. Importantly, there was no significant difference between groups in the 3-item composite score during baseline week −1 (Supplemental Table S1).

**Table 2.** Proportion of participants demonstrating an improvement at the end of the 6-week supplementation period for GI symptoms not included in the composite score.

Measure	Statistic/Category	BS50	Placebo
Abdominal cramping	Improvement	5 (13.2%)	2 (5.6%)
	No improvement	33 (86.8%)	34 (94.4%)
	OR (95% CI)	2.6 (0.5, 14.2)	
Reflux/ heartburn	Improvement	5 (13.2%)	3 (8.3%)
	No improvement	33 (86.8%)	33 (91.7%)
	OR (95% CI)	1.7 (0.4, 7.5)	
Borborygmus/ stomach rumbling	Improvement	16 (42.1%)	10 (27.8%)
	No improvement	22 (57.9%)	26 (72.2%)
	OR (95% CI)	1.9 (0.7, 5.0)	
Nausea	Improvement	0 (0.0%)	3 (8.3%)
	No improvement	38 (100.0%)	33 (91.7%)
	OR (95% CI)	n/a	
Vomiting	Improvement	n/a	n/a
	No improvement	n/a	n/a
	OR (95% CI)	n/a	

Values are model derived estimate (95% confidence interval)

The proportion of participants that had an improvement in abdominal cramping, reflux/heartburn, and boborygmus/stomach were compared between test products with the Chi-square test.

No nausea was reported for the BS50 group, thus, it was not possible to calculate odds ratio.

No participants reported vomiting at baseline and thus, it was not possible to calculate improvement.

CI, confidence interval; OR, odds ratio; n/a, not applicable

### Bowel habits

On average, study participants had 8 to 9 bowel movements per week, none to mild discomfort, straining or feelings of incomplete evacuation during bowel movement, and normal stool consistency (3 or 4 on the BSS). The change in the number of bowel movements over time was not significantly different (p = .94). The number of bowel movements increased in subsequent weeks, but the increase was small and was not significantly different between study products (Supplemental Table S2). The change in stool consistency over time was also not significantly different (p = .29) (Table 3). There were no significant changes from baseline and between study products for ratings of discomfort during bowel movement, straining during bowel movement, or feeling of incomplete evacuation (Supplemental Table S3).

**Table 3.** Weekly average stool consistency before and during the 6-week supplementation period.

Week	BS50	Placebo
−1	3.9 (3.6, 4.1)	3.6 (3.3, 3.8)
1	3.7 (3.5, 4.0)	3.7 (3.4, 4.0)
2	3.8 (3.5, 4.0)	3.7 (3.4, 3.9)
3	3.8 (3.5, 4.0)	3.8 (3.5, 4.1)
4	3.7 (3.5, 4.0)	4.0 (3.7, 4.2)
5	3.7 (3.4, 4.0)	3.7 (3.5, 4.0)
6	3.8 (3.5, 4.1)	3.7 (3.4, 4.0)

Values are model derived estimate (95% confidence interval)

The weekly average stool consistency was analyzed with a repeated measures model. The within group change from baseline was estimated along with a 95% confidence interval. Estimate statements were used to compare the change from baseline between groups and were estimated along with a 95% confidence interval. There were no significant differences within and between study products.

### Sleep and respiratory infection

At baseline, the average sleep quality was 7, indicating good sleep quality and > 75% reported having good to excellent sleep quality. Consumption of BS50 or placebo did not result in changes in sleep quality or the proportion of participants reporting good to excellent sleep quality (data not shown). Less than 11% of participants reported any respiratory infections (data not shown). Although analysis of respiratory infection episode was planned, the number of participants reporting any respiratory issues over the 6-week intervention was too low to provide any meaningful comparisons.

### Biochemical markers

Plasma lipids (TG, total-C, HDL-C, LDL-C), intestinal permeability markers (LBP, occludin, and zonulin), and inflammation markers (CRP, IFN-γ, IL-6, IL-10, IL-8, and TNF-α) were not affected by either study products (Table 4). There is a suggested distributional difference in the change from baseline (day –1) to end of week 6 (day 42) in IFN-γ, however, differences are small and likely to be clinically insignificant. Additionally, an increase in the circulating anti-inflammatory cytokine IL-10 in the BS50 group compared to placebo was suggested (p = .13 for ITT; p = .047 for PP).

**Table 4.** Plasma lipids, intestinal permeability markers, and inflammation markers at baseline (day –1) and end of the supplementation period (day 42).

Analyte	BS50	BS50	Placebo	Placebo
	day –1	Day 42	day –1	Day 42
HDL-C (mg/dL)	60.0 (24.3)	60.5 (22.4)	61.1 (18.7)	60.9 (18.8)
LDL-C (mg/dL)	113.2 (30.6)	113.9 (31.0)	117.9 (41.6)	116.1 (39.7)
Total-C (mg/dL)	197.6 (38.5)	198.9 (35.9)	197.9 (45.1)	196.9 (40.9)
TG (mg/dL)	136.1 (98.4)	137.2 (103.6)	103.8 (87.5)	109.5 (78.8)
LBP (ng/mL)	31.6 (5.7)	33.8 (5.4)	30.1 (7.4)	31.3 (7.2)
Occludin (ng/mL)	0.2 (0.1)	0.2 (0.1)	0.3 (0.1)	0.3 (0.1)
Zonulin (ng/mL)	1.3 (0.6)	1.2 (0.6)	1.4 (0.5)	1.4 (0.6)
CRP (mg/L)	27.3 (30.3)	27.4 (34.5)	19.8 (22.1)	32.3 (41.3)
IFN-γ (ng/mL)	1.0 (0.8)	1.1 (0.7)	0.9 (0.3)	1.0 (0.7)
IL-10 (pg/mL)	64.0 (19.9)	66.9 (17.6)	70.6 (36.4)	69.6 (36.1)
IL-6 (pg/mL)	76.4 (18.4)	78.5 (21.9)	79.8 (22.3)	82.9 (23.5)
IL-8 (pg/mL)	192.2 (38.1)	196.7 (34.3)	212.4 (93.8)	211.9 (97.8)
TNF-α (pg/mL)	153.1 (44.2)	168.4 (90.6)	143.0 (18.0)	143.1 (22.6)

Values are mean (standard deviation).

Analyzed using analysis of covariance (ANCOVA) approach. The change from baseline was used as the outcome and adjusted for baseline, product, sex, and BMI group. There were no difference within and between study products.

CRP, C-reactive protein; HDL-C, high density lipoprotein-cholesterol; LBP, lipopolysaccharide binding protein; LDL-C, low-density lipoprotein-cholesterol; total-C, total cholesterol; TG, triglyceride

### Safety

Consumption of BS50 was not associated with any clinically relevant changes in safety laboratory values. There were no changes in vital signs and body weight (data not shown) and there were also no safety concerns based on the fasting clinical chemistry and hematology (Supplemental Tables S4 and S5). Significant changes from baseline were detected for both study products for albumin (FDR q < .001), following placebo for albumin/globulin ratio (FDR q < .001). However, these changes are minor and subsequent analysis showed that these changes were not significantly different between study products (p = .59 and p = .45, respectively). A total of five AEs (four mild and one moderate) were reported by four participants. Of these, four were judged by the study physician as not related to study product. The remaining AE was judged as being possibly related to the study product whereby the participant who was in the placebo group reported experiencing daily headaches while on the product which ceased immediately once product consumption was stopped.

## Discussion

This is the first clinical trial describing the efficacy of *Bacillus subtilis* BS50 in relieving abdominal bloating and burping symptoms in healthy adults without FGIDs. The results demonstrate that daily supplementation of 2 × 10^9^ CFU BS50 per day for 6 weeks increased the proportion of participants showing improvement in the composite score for abdominal bloating, burping, and flatulence. BS50 also improved the individual symptoms of bloating and burping at 6 weeks compared to placebo. The effects of BS50 on improving GI symptoms are consistent with those observed in studies on other *Bacillaceae* strains. Daily supplementation for 4 weeks with a dietary supplement that contained 2 × 10^9^ CFU *W. coagulans* GBI-30, 6086 per serving in 61 participants with postprandial gas-related symptoms at baseline significantly improved Gastrointestinal Symptoms Rating Scale (GSRS) total scores and near-significantly improved GSRS abdominal distention sub-scores without significantly affecting GSRS flatus sub-score and Severity of Dyspepsia Assessment gas and bloating sub-scores.^34^ Penet et al. reported reductions in bloating intensity, number of days with abdominal discomfort, gas, and bloating, and duration of gas, compared to placebo following daily supplementation with *B. subtilis* MB40 at 5 × 10^9^ CFU/day for 4 weeks in 100 healthy adults.^39^ This clinically meaningful reduction was only observed in the male subgroup. In our analysis of the 3-item composite GI score, the interaction term between treatment and sex was not significant, suggesting that the beneficial effect of BS50 following 6 weeks of daily supplementation on GI symptoms was not dependent on sex. Daily supplementation of either *B. subtilis* R0179 up to 10 × 10^9^ CFU/day or *B. inaquosorum* DE111 (formerly *B. subtilis* subsp. *inaquosorum* DE111; 2.5 × 10^9^ CFU/day) for 4 weeks showed no difference in participant self-reported GI symptoms compared to placebo groups.^50,51^ It is noteworthy that the aforementioned studies and ours were dissimilar on several aspects that may contribute to the contrast in results. These include differences in supplementation length (4 weeks vs. 6 weeks), questionnaires used, as well as definition and analysis of efficacy outcomes (odds ratio vs. mean scores). Additionally, we required our study products to be consumed with a meal whereas the consumption of the probiotic spore with meals was not specified in some of the prior studies. At least 6 weeks of daily supplementation with a meal is recommended for clinical trials of spore-forming strains with a primary outcome related to abdominal bloating because consumption with a meal is expected to critically promote growth of the spores and release of bioactive molecules such as antimicrobials and digestive enzymes.^16^

Although the exact mechanism by which BS50 improved GI symptoms remains unknown, it is likely dependent on the transition of BS50 spores to vegetative cells in the stomach and small intestine. *Bacillaceae* spores, including *B. subtilis*, have previously been detected in human fecal and ileal biopsy samples in numerous studies, suggesting that spores naturally occur and germinate in the human intestine.^52–55^ Additionally, Colom et al. generated compelling *in vivo* evidence that oral administration of *B. inaquosorum* DE111 spores to ileostomy patients yields spore germination and vegetative bacterial cells across 8 hours of postprandial ileal effluent sampling.^16^ Vegetative BS50 has been shown to secrete digestive enzymes *in vitro* (Supplemental Table S6). Taken together, BS50 may germinate in the intestine and secrete enzymes that help digest food and improve nutrient absorption, theoretically leading to less fermentation and gas production in the lower intestine. Consistent with this hypothesis, the postprandial plasma concentration of several amino acids was elevated following consumption of pea protein with a 10 × 10^9^ CFU dose of *Lacticaseibacillus paracasei* in an acute aminoacidemia trial following 2 weeks of daily supplementation, compared to the maltodextrin placebo.^56^ Similarly, a 1 × 10^9^ CFU dose of *W. coagulans* GBI-30, 6086 with milk protein concentrate daily for 2 weeks increased postprandial plasma amino acid concentrations compared to milk protein concentrate alone.^40^ Separately, 2 × 10^9^ CFU/day supplementation with *W. coagulans* Unique IS-2 with whey protein for 60 days increased fasting plasma concentrations of amino acids.^41^

An extension of these observations is that probiotics such as BS50 may indirectly affect nutrient-sensing through increasing intestinal nutrient concentrations, promoting satiety, and lowering food consumption, thus lessening the chance of bloating associated with overconsumption. As the satiety hormones glucagon-like peptide 1 (GLP-1) and peptide YY (PYY) are implicated in nutrient sensing, it is intriguing that a multi-ingredient dietary supplement containing 2 × 10^9^ CFU *B. subtilis* DSM 32315 reduced fasting plasma GLP-1 and PYY concentrations by 36% and 40%, respectively, after 4 weeks of daily supplementation.^57^ GLP-1 is synthesized and released by enteroendocrine L cells of the intestine in response to digestion and can also be measured in the bloodstream.^58^ Circulating GLP-1 concentrations were directly correlated with metabolic syndrome and obesity in a study of 140 female participants.^59^ However, it is the acute response and contribution of GLP-1 to insulin regulation that is traditionally studied in oral glucose and meal challenges. In this context, the probiotic strains *Limosilactobacillus reuteri* SD5865 (formerly *Lactobacillus reuteri*) and *Anaerobutyricum soehngenii* CH106 have been shown to beneficially increase acute postprandial GLP-1 response following oral supplementation and duodenal infusion, respectively, in clinical trials,^60,61^ as well as other strains in animal studies.^62,63^ Many clinical trials of probiotic supplementation have shown direct benefits on blood glucose control in patients with diabetes.^64^ When our participants were categorized as having normal (< 100 mg/dL) or elevated (≥ 100 mg/dL) glucose, seven out of 36 (19%) participants in the placebo group who initially had normal fasting glucose levels ended the study with elevated levels. This occurred in only four out of 28 (14%) of participants in the BS50 group. Furthermore, of the 10 participants randomized to the BS50 group who had elevated glucose at baseline, six ended the study with normal glucose levels. Although this study was not designed to assess the effects of BS50 on blood glucose, the results imply that BS50 may beneficially regulate blood glucose and thus, more research is needed to understand the effects of *Bacillaceae* strains and their secreted molecules on GLP-1 regulation and blood glucose control.

We did not observe any effects of BS50 on the number of bowel movements, stool consistency, and bowel movement-related symptoms following 6 weeks of supplementation. This is consistent with a previous study of *B. inaquosorum* DE111 whereby supplementation at 5 × 10^9^ CFU/day for 20 days did not affect stool consistency or the number of bowel movements in healthy adults.^65^ These null results are likely due to the abbreviated duration of supplementation and participants having healthy and normal bowel movements and stool consistency as well as the low occurrence of discomfort, straining, or feelings of incomplete evacuation during bowel movement at baseline. In contrast, adults suffering from occasional constipation and/or diarrhea demonstrated improvements in stool consistency following 15 weeks of supplementation with 1 × 10^9^ CFU/day *B. inaquosorum* DE111 compared to placebo.^66^ Additionally, supplementation with *B. subtilis* C-3102 at 2.2 × 10^9^ CFU/day for 8 weeks decreased fecal water content and beneficially increased BSS scores in adults with loose stools (BSS 5, 6, or 7).^32^ To better understand the effects of BS50 in improving bowel movement and stool consistency, additional clinical investigations across at least 8 weeks of supplementation in populations with constipation, diarrhea, and other stool-related abnormalities are warranted. Such an approach is consistent with published studies showing improvement of GI symptoms or stool characteristics following at least 8 weeks of *B. subtilis* or *W. coagulans* supplementation in patients with IBS.^30,31,33,35,37,67^

An increase in the circulating anti-inflammatory cytokine IL-10 in the BS50 group compared to placebo was suggested (p = .047, PP population). IL-10 is a robust anti-inflammatory and immunosuppressive cytokine protein produced and secreted by many immune cells, including monocytes, macrophages, regulatory T cells, and T helper type 2 cells.^68^ IL-10 plays a role in dampening inflammatory responses by reducing antigen presentation and inhibiting the release of pro-inflammatory cytokines.^69^ Lower circulating and intestinal mucosal levels of IL-10 are associated with IBS,^70–72^ so IL-10 has become a target for upregulation in IBS. Oral supplementation of *Lactobacillaceae* and *Bifidobacterium* strains have previously been associated with increased circulating concentrations of IL-10 in clinical trials;^73–77^ however, the mechanism is not well understood. It is possible that probiotic bacteria or secreted molecules interact directly with the intestinal epithelium to modulate systemic inflammation, or indirectly through modulation of the intestinal microbiota to lessen the abundance and impact of pro-inflammatory or pathogenic strains.^14,78^ In support, *in vitro* studies suggest possible antimicrobial activities of BS50 (Supplemental Table S7). Future clinical studies of BS50 will aim to better describe effects on intestinal inflammation and microbiota composition.

We did not observe any significant changes in plasma lipids or inflammation markers. These results are not unexpected as this study was not powered to assess these markers nor was the effort made to recruit participants with increased lipids or inflammation. While the beneficial effects of other strains of probiotics on plasma lipids and inflammation have been demonstrated,^79,80^ Trotter et al. reported that *B. inaquosorum* DE111 at 1 × 10^9^ CFU/day for 4 weeks reduced total-C and non-HDL-C compared to baseline, but not when compared to placebo in healthy adults. In that study, total-C and non-HDL-C decreased in all treatment groups, including the placebo group, albeit not significantly.^81^ Thus, the decreases in plasma lipids may be unrelated to probiotic supplementation. A study on *B. inaquosorum* DE111 in healthy adults also reported no effects on CRP.^65^ Future studies of *Bacillaceae* strains such as BS50 will likely require greater participant enrollment to better understand whether immune cells and inflammation play a role in reducing GI symptoms.

Another important objective of this study was to evaluate the safety of BS50. Overall, BS50 did not adversely affect blood chemistry or hematology, blood pressure, or body weight. Only one adverse event possibly related to study product was reported by one participant who was in the placebo group. Additionally, there were no changes or significant increases in any of the GI symptoms assessed, nor were there any concerns related to changes in plasma lipid, inflammation markers, intestinal permeability markers, sleep, or rate of respiratory infection. Thus, the current results indicate that BS50 at 2 × 10^9^ CFU/day up to 6 weeks is safe in healthy adults.

## Conclusion

Results from this first-in-human study of the probiotic strain *Bacillus subtilis* BS50 demonstrate that daily supplementation at 2 × 10^9^ CFU/day increased the proportion of participants showing improvement in the composite score for bloating, burping, and flatulence, compared to placebo. BS50 also improved the individual symptoms of bloating and burping across the 6 weeks of daily probiotic supplementation. In contrast to similar studies of *Bacillaceae* strains, we conducted this trial in exclusively health adults without any history of functional GI disorders such as IBS, dyspepsia, gastroesophageal reflux disease, constipation, and diarrhea. These results suggest that *B. subtilis* BS50 supplementation is a well-tolerated and safe probiotic approach to improve digestive health, support GI comfort, and alleviate gas-related GI symptoms in healthy adults.

## Supplementary Material

Supplemental Material

## Data Availability

The data presented in this study are available upon reasonable request from the corresponding author.
