# Supplementary material for: The probiotic Bacillus subtilis BS50 decreases gastrointestinal symptoms in healthy adults: a randomized, double-blind, placebo-controlled trial
Source: Gut Microbes. 2022 Oct 21;14(1):2122668. doi: 10.1080/19490976.2022.2122668 (PMC9590435; doi:10.1080/19490976.2022.2122668)
Supplement: Supplemental Material [file KGMI_A_2122668_SM8169.pdf]

SUPPLEMENTARY MATERIAL FOR:

The Probiotic *Bacillus subtilis* BS50 Decreases Gastrointestinal Symptoms in Healthy Adults: A Randomized, Double-Blind, Placebo-Controlled Trial

Sean M. Garvey[a,b,*], Eunice Mah[c], Traci M. Blonquist[c], Valerie N. Kaden[c], and Jessica L. Spears[a,*]

[a]BIO-CAT Microbials, LLC, Shakopee, MN, USA; [b]BIO-CAT, Inc., Troy, VA, USA; [c]Biofortis Research, Inc., Addison, IL, USA

*Corresponding authors:

Jessica L. Spears
JSpears@bio-cat.com
Department of Research and Development, BIO-CAT Microbials, LLC, 689 Canterbury Rd S, Shakopee, MN 55379, USA

Sean M. Garvey
SGarvey@bio-cat.com
Department of Research and Development, BIO-CAT, Inc., 9117 Three Notch Rd, Troy, VA 22974, USA

Supplemental Table 1. Weekly 3-item composite score

Week	Source	Statistic	BS50	Placebo
−1	Summary statistics	n	38	38
		Median (Range)	16.0 (3.0, 30.0)	11.0 (5.0, 34.0)
		IQR Limits	9.0, 18.0	8.0, 15.0
		Mean (SD)	14.6 (6.9)	12.4 (6.1)
	Model derived[1]	Estimate (95% CI)	14.7 (12.5, 16.8)	12.6 (10.4, 14.8)
		Difference Estimate (95% CI)	2.0 (-1.0, 5.1)	
1	Summary statistics	n	36	36
		Median (Range)	14.0 (5.0, 35.0)	13.0 (1.0, 27.0)
		IQR Limits	8.7, 19.0	7.0, 15.5
		Mean (SD)	14.9 (7.3)	12.6 (5.9)
	Model derived	Estimate (95% CI)	15.0 (12.8, 17.2)	12.8 (10.6, 15.0)
		Difference Estimate (95% CI)	2.2 (-0.9, 5.3)	
		Change Estimate (95% CI)	0.3 (-2.4, 3.0)	0.2 (-2.5, 2.8)
		Difference Change Estimate (95% CI)	0.2 (-3.6, 3.9)	
		p-value	.94	
2	Summary statistics	n	38	38
		Median (Range)	14.0 (3.0, 32.0)	12.0 (1.4, 30.0)
		IQR Limits	10.0, 19.0	7.0, 14.0
		Mean (SD)	14.5 (6.6)	11.5 (6.2)
	Model derived	Estimate (95% CI)	14.6 (12.4, 16.8)	11.7 (9.5, 13.9)
		Difference Estimate (95% CI)	2.9 (-0.2, 6.0)	
		Change Estimate (95% CI)	-0.0 (-2.6, 2.5)	-0.9 (-3.4, 1.6)
		Difference Change Estimate (95% CI)	0.9 (-2.7, 4.4)	
		p-value	.64	
3	Summary statistics	n	38	38
		Median (Range)	14.0 (4.0, 34.0)	11.8 (1.0, 35.0)
		IQR Limits	10.0, 19.0	7.0, 15.0

Week	Source	Statistic	BS50	Placebo
		Mean (SD)	14.9 (7.0)	12.0 (6.9)
	Model derived	Estimate (95% CI)	15.0 (12.8, 17.2)	12.2 (10.0, 14.4)
		Difference Estimate (95% CI)	2.8 (-0.2, 5.9)	
		Change Estimate (95% CI)	0.4 (-2.0, 2.7)	-0.4 (-2.8, 2.0)
		Difference Change Estimate (95% CI)	0.8 (-2.6, 4.2)	
		p-value	.64	
4	Summary statistics	n	38	38
		Median (Range)	14.0 (3.0, 33.0)	12.0 (0.0, 35.0)
		IQR Limits	10.0, 18.0	7.0, 14.0
		Mean (SD)	14.5 (6.7)	11.5 (6.9)
	Model derived	Estimate (95% CI)	14.6 (12.4, 16.7)	11.7 (9.5, 13.8)
		Difference Estimate (95% CI)	2.9 (-0.2, 6.0)	
		Change Estimate (95% CI)	-0.1 (-2.3, 2.1)	-1.0 (-3.1, 1.2)
		Difference Change Estimate (95% CI)	0.9 (-2.2, 3.9)	
		p-value	.58	
5	Summary statistics	n	38	38
		Median (Range)	14.0 (0.0, 32.0)	12.0 (0.0, 35.0)
		IQR Limits	9.3, 20.0	7.0, 14.0
		Mean (SD)	14.4 (8.1)	11.8 (6.5)
	Model derived	Estimate (95% CI)	14.5 (12.3, 16.7)	12.0 (9.8, 14.2)
		Difference Estimate (95% CI)	2.5 (-0.6, 5.6)	
		Change Estimate (95% CI)	-0.2 (-2.0, 1.7)	-0.6 (-2.5, 1.2)
		Difference Change Estimate (95% CI)	0.5 (-2.1, 3.1)	
		p-value	.72	
6	Summary statistics	n	38	38
		Median (Range)	14.0 (1.2, 41.0)	14.0 (0.0, 35.0)
		IQR Limits	8.0, 17.0	7.0, 16.0
		Mean (SD)	13.5 (8.0)	12.8 (7.2)

Week	Source	Statistic	BS50	Placebo
	Model derived	Estimate (95% CI)	13.6 (11.4, 15.7)	13.0 (10.8, 15.2)
		Difference Estimate (95% CI)	0.5 (-2.5, 3.6)	
		Change Estimate (95% CI)	-1.1 (-2.5, 0.3)	0.4 (-1.0, 1.8)
		Difference Change Estimate (95% CI)	-1.5 (-3.5, 0.5)	
		p-value	.14	

Abbreviations: CI, confidence interval; IQR, interquartile range; n, sample size; SD, standard deviation

Footnotes:

Analyzed with a repeated measures model where the within subject correlation was modelled with an AR(1) covariance structure. The model contained fixed effect terms for product, week, product × week interaction, sex, and BMI group.

Estimate statements were used to estimate the within product change from baseline (week −1) to each follow-up time point as well as the difference (95% confidence interval) between products in the change from baseline to each follow-up time.

Supplemental Table 2. Weekly number of bowel movements

Week	Source	Statistic	BS50	Placebo
−1	Summary statistics	n	38	38
		Median (Range)	8.0 (3.0, 21.0)	7.0 (3.0, 20.0)
		IQR Limits	6.0, 11.0	6.0, 9.0
		Mean (SD)	8.6 (3.6)	7.9 (3.1)
	Model derived	Estimate (95% CI)	8.6 (7.7, 9.7)	7.9 (7.0, 9.0)
		Difference Estimate (95% CI)	1.1 (0.9, 1.3)	
1	Summary statistics	n	38	37
		Median (Range)	9.0 (4.0, 20.0)	9.0 (4.0, 16.0)
		IQR Limits	7.0, 12.0	7.0, 11.0
		Mean (SD)	9.7 (3.2)	9.3 (2.5)
	Model derived	Estimate (95% CI)	9.7 (8.7, 10.8)	9.2 (8.3, 10.4)
		Difference Estimate (95% CI)[2]	1.0 (0.9, 1.2)	
		Change Estimate (95% CI)[3]	1.1 (1.0, 1.3)	1.2 (1.0, 1.4)
		Difference Change Estimate (95% CI)	1.0 (0.8, 1.2)	
		p-value[4]	.77	
2	Summary statistics	n	38	37
		Median (Range)	9.0 (4.0, 24.0)	9.0 (3.0, 18.0)
		IQR Limits	7.0, 11.0	8.0, 10.0
		Mean (SD)	9.6 (4.2)	9.2 (3.0)
	Model derived	Estimate (95% CI)	9.6 (8.6, 10.7)	9.2 (8.2, 10.3)
		Difference Estimate (95% CI)[2]	1.0 (0.9, 1.2)	
		Change Estimate (95% CI)[3]	1.1 (1.0, 1.3)	1.2 (1.0, 1.3)
		Difference Change Estimate (95% CI)	1.0 (0.8, 1.2)	
		p-value[4]	.75	
3	Summary statistics	n	37	37
		Median (Range)	8.0 (4.0, 18.0)	8.0 (3.0, 14.0)
		IQR Limits	7.0, 11.0	7.0, 10.0

Week	Source	Statistic	BS50	Placebo
		Mean (SD)	9.3 (3.6)	8.6 (2.4)
	Model derived	Estimate (95% CI)	9.2 (8.3, 10.3)	8.5 (7.6, 9.6)
		Difference Estimate (95% CI)[2]	1.1 (0.9, 1.3)	
		Change Estimate (95% CI)[3]	1.1 (0.9, 1.2)	1.1 (0.9, 1.2)
		Difference Change Estimate (95% CI)	1.0 (0.8, 1.2)	
		p-value[4]	.99	
4	Summary statistics	n	38	37
		Median (Range)	8.0 (4.0, 20.0)	8.0 (3.0, 13.0)
		IQR Limits	7.0, 12.0	7.0, 10.0
		Mean (SD)	9.4 (3.8)	8.5 (2.5)
	Model derived	Estimate (95% CI)	9.4 (8.4, 10.5)	8.5 (7.5, 9.6)
		Difference Estimate (95% CI)[2]	1.1 (0.9, 1.3)	
		Change Estimate (95% CI)[3]	1.1 (1.0, 1.2)	1.1 (0.9, 1.2)
		Difference Change Estimate (95% CI)	1.0 (0.8, 1.2)	
		p-value[4]	.85	
5	Summary statistics	n	38	38
		Median (Range)	8.0 (5.0, 19.0)	8.0 (4.0, 16.0)
		IQR Limits	7.0, 12.0	7.0, 10.0
		Mean (SD)	9.3 (3.6)	8.5 (2.6)
	Model derived	Estimate (95% CI)	9.2 (8.2, 10.3)	8.5 (7.5, 9.5)
		Difference Estimate (95% CI)[2]	1.1 (0.9, 1.3)	
		Change Estimate (95% CI)[3]	1.1 (1.0, 1.2)	1.1 (0.9, 1.2)
		Difference Change Estimate (95% CI)	1.0 (0.9, 1.2)	
		p-value[4]	.94	
6	Summary statistics	n	38	36
		Median (Range)	7.0 (3.0, 23.0)	9.0 (6.0, 15.0)
		IQR Limits	7.0, 12.0	7.0, 11.0
		Mean (SD)	9.4 (4.2)	9.3 (2.4)

Week	Source	Statistic	BS50	Placebo
	Model derived	Estimate (95% CI)	9.4 (8.4, 10.5)	9.2 (8.2, 10.3)
		Difference Estimate (95% CI)[2]	1.0 (0.9, 1.2)	
		Change Estimate (95% CI)[3]	1.1 (1.0, 1.2)	1.2 (1.1, 1.3)
		Difference Change Estimate (95% CI)	0.9 (0.8, 1.1)	
		p-value[4]	.42	

Abbreviations: CI, confidence interval; IQR, interquartile range; n, sample size; SD, standard deviation

Footnotes:

[1]Weekly bowel movement imputed if at least 4 of 7 days were reported in a given week. Analyzed with a generalized linear mixed model following the Poisson distribution with a log link.

[2]Difference between products presented as a ratio

[3]Change between week and baseline (week −1) presented as a ratio

[4]p-value is model derived from the difference in the change from baseline (week −1)

Supplemental Table 3. Average rating of symptoms experienced during bowel movements

Symptom	Week	BS50	Placebo
Discomfort	−1	0.0 (0.0, 1.7)	0.1 (0.0, 1.6)
	1	0.2 (0.0, 1.5)	0.2 (0.0, 2.0)
	2	0.2 (0.0, 1.2)	0.2 (0.0, 1.6)
	3	0.2 (0.0, 1.4)	0.2 (0.0, 1.5)
	4	0.2 (0.0, 1.5)	0.1 (0.0, 1.9)
	5	0.0 (0.0, 1.6)	0.1 (0.0, 1.6)
	6	0.2 (0.0, 1.5)	0.0 (0.0, 1.9)
Incomplete evacuation	−1	0.2 (0.0, 1.8)	0.2 (0.0, 1.6)
	1	0.2 (0.0, 1.8)	0.1 (0.0, 1.5)
	2	0.2 (0.0, 1.7)	0.1 (0.0, 1.6)
	3	0.2 (0.0, 2.2)	0.1 (0.0, 1.5)
	4	0.2 (0.0, 1.6)	0.0 (0.0, 1.5)
	5	0.2 (0.0, 1.5)	0.0 (0.0, 1.6)
	6	0.1 (0.0, 2.2)	0.0 (0.0, 1.9)
Straining	−1	0.3 (0.0, 1.9)	0.3 (0.0, 1.7)
	1	0.4 (0.0, 1.5)	0.2 (0.0, 2.0)
	2	0.4 (0.0, 1.3)	0.3 (0.0, 1.6)
	3	0.2 (0.0, 1.8)	0.2 (0.0, 1.5)
	4	0.3 (0.0, 1.5)	0.1 (0.0, 1.9)
	5	0.1 (0.0, 2.0)	0.1 (0.0, 1.6)
	6	0.2 (0.0, 1.7)	0.0 (0.0, 1.8)

Abbreviations: CI, confidence interval; IQR, interquartile range; n, sample size; SD, standard deviation

Footnotes:

Stratified Wilcoxon (Van Elteren Test) was performed adjusting for sex and BMI group.

There were no significant differences within and between study products.

Supplemental Table 4. Fasting clinical chemistry

Test	Statistic	Day −7		Day 42		Change	
		BS50	Placebo	BS50	Placebo	BS50	Placebo
Albumin / Globulin Ratio	n	38	38	38	38	38	38
	IQR Limits	1.1, 1.3	1.0, 1.2	1.0, 1.2	1.0, 1.1	-0.1, 0.0	-0.1, 0.0
	Mean (SD)	1.1 (0.2)	1.1 (0.1)	1.1 (0.2)	1.0 (0.1)	-0.1 (0.1)	-0.1 (0.1)
	Median (Range)	1.1 (0.7, 1.5)	1.1 (0.9, 1.4)	1.1 (0.8, 1.5)	1.0 (0.8, 1.3)	-0.1 (-0.3, 0.2)	-0.1 (-0.3, 0.1)
	Wilcoxon signed rank p-value[1]					.006	< .001
	FDR q-value[1]					.093	< .001
	Wilcoxon rank sum p-value[1]					.45	
Albumin (g/dL)	n	38	38	38	38	38	38
	IQR Limits	3.7, 4.1	3.8, 4.1	3.5, 4.0	3.5, 3.9	-0.3, -0.1	-0.3, -0.1
	Mean (SD)	3.9 (0.3)	3.9 (0.3)	3.7 (0.3)	3.7 (0.3)	-0.2 (0.2)	-0.2 (0.2)
	Median (Range)	3.9 (3.2, 4.5)	4.0 (3.2, 4.5)	3.7 (2.9, 4.3)	3.8 (2.8, 4.2)	-0.2 (-0.7, 0.3)	-0.2 (-0.5, 0.4)
	Wilcoxon signed rank p-value					< .001	< .001
	FDR q-value					< .001	< .001
	Wilcoxon rank sum p-value					.59	
Alkaline Phosphatase (U/L)	n	38	38	38	38	38	38
	IQR Limits	52.0, 76.0	49.0, 74.0	49.0, 79.0	53.0, 77.0	-5.0, 4.0	-4.0, 4.0
	Mean (SD)	63.7 (16.3)	64.1 (16.8)	63.3 (18.1)	64.7 (16.8)	-0.4 (7.5)	0.7 (7.7)

Test	Statistic	Day −7		Day 42		Change	
		BS50	Placebo	BS50	Placebo	BS50	Placebo
	Median (Range)	65.0 (27.0, 94.0)	65.5 (32.0, 103.0)	62.0 (28.0, 95.0)	65.5 (37.0, 104.0)	2.0 (-17.0, 11.0)	0.0 (-22.0, 23.0)
	Wilcoxon signed rank p-value					.92	.62
	FDR q-value					.96	.78
Alanine Aminotransferase (U/L)	n	38	38	38	38	38	38
	IQR Limits	21.0, 33.0	22.0, 37.0	20.0, 38.0	21.0, 32.0	-5.0, 2.0	-6.0, 1.0
	Mean (SD)	31.8 (15.6)	30.8 (10.2)	30.3 (14.8)	30.5 (15.4)	-1.6 (7.5)	-0.4 (11.6)
	Median (Range)	25.0 (17.0, 70.0)	27.0 (18.0, 65.0)	26.0 (13.0, 80.0)	25.5 (15.0, 92.0)	-2.0 (-23.0, 19.0)	-2.5 (-19.0, 50.0)
	Wilcoxon signed rank p-value					.064	.060
	FDR q-value					.31	.31
Anion Gap (mmol/L)	n	38	38	38	38	38	38
	IQR Limits	3.0, 6.0	3.0, 6.0	3.0, 6.0	3.0, 6.0	-2.0, 2.0	-2.0, 2.0
	Mean (SD)	4.6 (1.4)	4.6 (1.8)	4.8 (2.0)	4.9 (2.0)	0.3 (2.1)	0.3 (2.4)
	Median (Range)	4.5 (2.0, 7.0)	4.0 (1.0, 8.0)	5.0 (1.0, 10.0)	5.0 (1.0, 9.0)	1.0 (-4.0, 3.0)	1.0 (-5.0, 6.0)
	Wilcoxon signed rank p-value					.45	.54
	FDR q-value					.78	.78
Aspartate Aminotransferase (U/L)	n	38	38	38	38	38	38

Test	Statistic	Day −7		Day 42		Change	
		BS50	Placebo	BS50	Placebo	BS50	Placebo
	IQR Limits	16.0, 24.0	17.0, 23.0	16.0, 25.0	17.0, 22.0	-2.0, 3.0	-3.0, 2.0
	Mean (SD)	20.4 (6.3)	19.9 (4.9)	20.6 (6.2)	19.8 (5.2)	0.2 (4.4)	-0.1 (4.8)
	Median (Range)	19.0 (11.0, 40.0)	19.5 (6.0, 29.0)	19.0 (12.0, 35.0)	19.0 (10.0, 34.0)	0.0 (-16.0, 9.0)	-0.5 (-11.0, 13.0)
	Wilcoxon signed rank p-value					.48	.67
	FDR q-value					.78	.80
Total Bilirubin (mg/dL)	n	38	38	38	38	38	38
	IQR Limits	0.5, 0.7	0.5, 0.8	0.5, 0.7	0.4, 0.8	-0.1, 0.0	-0.2, 0.1
	Mean (SD)	0.6 (0.2)	0.7 (0.3)	0.6 (0.3)	0.6 (0.3)	-0.0 (0.2)	-0.0 (0.3)
	Median (Range)	0.6 (0.3, 1.4)	0.6 (0.3, 1.3)	0.6 (0.2, 2.0)	0.6 (0.2, 1.9)	-0.1 (-0.3, 1.0)	-0.1 (-0.8, 0.7)
	Wilcoxon signed rank p-value					.22	.15
	FDR q-value					.61	.46
Blood Urea Nitrogen (mg/dL)	n	38	38	38	38	38	38
	IQR Limits	11.0, 17.0	11.0, 18.0	12.0, 16.0	12.0, 20.0	-2.0, 2.0	-3.0, 3.0
	Mean (SD)	14.3 (4.2)	15.6 (4.6)	14.4 (3.3)	15.5 (4.9)	0.1 (3.6)	-0.1 (4.0)
	Median (Range)	13.5 (7.0, 25.0)	16.0 (8.0, 26.0)	14.0 (7.0, 24.0)	14.5 (9.0, 26.0)	1.0 (-9.0, 8.0)	0.0 (-6.0, 14.0)
	Wilcoxon signed rank p-value					.61	.61
	FDR q-value					.78	.78

Test	Statistic	Day −7		Day 42		Change	
		BS50	Placebo	BS50	Placebo	BS50	Placebo
Blood Urea Nitrogen /Creatinine Ratio	n	38	38	38	38	38	38
	IQR Limits	13.3, 20.4	15.2, 21.9	13.6, 21.0	14.9, 24.3	-2.1, 3.1	-4.0, 3.2
	Mean (SD)	17.2 (4.8)	19.6 (8.4)	17.8 (5.2)	19.4 (6.1)	0.6 (4.8)	-0.1 (6.9)
	Median (Range)	16.6 (8.9, 29.1)	18.8 (8.8, 56.9)	17.2 (7.9, 33.3)	18.1 (9.6, 32.5)	0.8 (-10.1, 16.9)	0.2 (-28.3, 17.5)
	Wilcoxon signed rank p-value					.56	.91
	FDR q-value					.78	.96
Total Calcium (mg/dL)	n	38	38	38	38	38	38
	IQR Limits	8.8, 9.3	8.9, 9.3	8.8, 9.3	9.0, 9.3	-0.3, 0.3	-0.2, 0.3
	Mean (SD)	9.1 (0.3)	9.1 (0.3)	9.1 (0.3)	9.1 (0.3)	-0.0 (0.4)	0.1 (0.4)
	Median (Range)	9.2 (8.4, 9.7)	9.0 (8.4, 9.8)	9.1 (8.4, 9.8)	9.1 (8.4, 9.9)	0.0 (-0.8, 0.8)	0.1 (-0.9, 0.9)
	Wilcoxon signed rank p-value					.71	.46
	FDR q-value					.84	.78
Calculated Osmolality (mOsm/kg)	n	38	38	38	38	38	38
	IQR Limits	286.0, 291.0	287.0, 294.0	287.0, 292.0	289.0, 294.0	-1.0, 3.0	-2.0, 4.0
	Mean (SD)	288.7 (4.1)	290.5 (4.6)	289.7 (4.4)	291.4 (4.3)	1.1 (3.4)	0.9 (4.2)
	Median (Range)	288.0 (280.0, 300.0)	290.0 (281.0, 301.0)	289.0 (279.0, 299.0)	290.5 (281.0, 303.0)	0.0 (-3.0, 14.0)	1.0 (-6.0, 10.0)

Test	Statistic	Day −7		Day 42		Change	
		BS50	Placebo	BS50	Placebo	BS50	Placebo
	Wilcoxon signed rank p-value					.12	.27
	FDR q-value					.38	.65
Chloride (mmol/L)	n	38	38	38	38	38	38
	IQR Limits	106.0, 108.0	107.0, 109.0	105.0, 108.0	106.0, 109.0	-1.0, 2.0	-1.0, 2.0
	Mean (SD)	106.9 (1.9)	107.9 (2.0)	107.3 (2.5)	108.1 (2.5)	0.4 (2.1)	0.2 (2.3)
	Median (Range)	107.0 (103.0, 111.0)	108.0 (103.0, 112.0)	107.0 (103.0, 114.0)	108.0 (103.0, 115.0)	0.0 (-4.0, 6.0)	0.0 (-6.0, 6.0)
	Wilcoxon signed rank p-value					.41	.53
	FDR q-value					.78	.78
CO_2 (mmol/L)	n	38	38	38	38	38	38
	IQR Limits	26.0, 29.0	26.0, 29.0	26.0, 29.0	26.0, 29.0	-1.0, 1.0	-1.0, 1.0
	Mean (SD)	27.7 (2.1)	27.4 (1.9)	27.6 (2.1)	27.4 (2.1)	-0.1 (1.8)	-0.1 (2.1)
	Median (Range)	27.5 (24.0, 32.0)	27.5 (24.0, 31.0)	28.0 (24.0, 32.0)	28.0 (23.0, 31.0)	0.0 (-3.0, 3.0)	0.0 (-4.0, 5.0)
	Wilcoxon signed rank p-value					.79	.90
	FDR q-value					.90	.96
Creatinine (mg/dL)	n	38	38	38	38	38	38
	IQR Limits	0.7, 1.0	0.7, 1.0	0.7, 1.0	0.7, 0.9	-0.1, 0.0	-0.1, 0.0
	Mean (SD)	0.8 (0.2)	0.9 (0.2)	0.8 (0.2)	0.8 (0.2)	-0.0 (0.1)	-0.0 (0.1)

Test	Statistic	Day −7		Day 42		Change	
		BS50	Placebo	BS50	Placebo	BS50	Placebo
	Median (Range)	0.9 (0.5, 1.2)	0.8 (0.5, 1.3)	0.8 (0.5, 1.1)	0.8 (0.5, 1.2)	-0.0 (-0.1, 0.2)	-0.0 (-0.2, 0.2)
	Wilcoxon signed rank p-value					.30	.006
	FDR q-value					.68	.093
	Wilcoxon rank sum p-value					.098	
Globulin (g/dL)	n	38	38	38	38	38	38
	IQR Limits	3.3, 3.8	3.3, 3.9	3.2, 3.8	3.3, 3.9	-0.2, 0.2	-0.1, 0.2
	Mean (SD)	3.5 (0.4)	3.6 (0.4)	3.5 (0.5)	3.6 (0.4)	0.0 (0.3)	0.1 (0.3)
	Median (Range)	3.6 (2.6, 4.3)	3.6 (2.8, 4.0)	3.5 (2.5, 4.5)	3.6 (2.8, 4.4)	0.0 (-1.1, 0.5)	0.0 (-0.5, 0.7)
	Wilcoxon signed rank p-value					.60	.10
	FDR q-value					.78	.36
Glucose (mg/dL)	n	38	38	38	38	38	38
	IQR Limits	86.0, 100.0	89.0, 96.0	87.0, 98.0	88.0, 99.0	-4.0, 3.0	-5.0, 6.0
	Mean (SD)	94.0 (9.6)	92.3 (7.3)	92.5 (11.3)	93.1 (6.9)	-1.6 (9.1)	0.7 (8.2)
	Median (Range)	93.0 (79.0, 119.0)	92.5 (73.0, 113.0)	92.0 (65.0, 130.0)	94.0 (74.0, 103.0)	1.0 (-46.0, 11.0)	0.0 (-17.0, 17.0)
	Wilcoxon signed rank p-value					.60	.64
	FDR q-value					.78	.79

Test	Statistic	Day −7		Day 42		Change	
		BS50	Placebo	BS50	Placebo	BS50	Placebo
Potassium (mmol/L)	n	38	38	38	38	38	38
	IQR Limits	4.1, 4.5	4.1, 4.7	4.1, 4.5	4.1, 4.7	-0.2, 0.2	-0.3, 0.3
	Mean (SD)	4.3 (0.4)	4.4 (0.4)	4.3 (0.3)	4.5 (0.5)	-0.1 (0.4)	0.1 (0.5)
	Median (Range)	4.3 (3.7, 5.4)	4.3 (3.9, 5.5)	4.3 (3.8, 4.8)	4.4 (3.5, 5.8)	-0.0 (-1.5, 0.6)	0.0 (-0.9, 1.5)
	Wilcoxon signed rank p-value					.28	.72
	FDR q-value					.65	.84
Sodium (mmol/L)	n	38	38	38	38	38	38
	IQR Limits	138.0, 140.0	139.0, 141.0	138.0, 141.0	139.0, 141.0	-1.0, 1.0	-1.0, 2.0
	Mean (SD)	139.2 (1.6)	139.9 (1.9)	139.7 (2.0)	140.3 (1.9)	0.5 (1.5)	0.5 (1.9)
	Median (Range)	139.0 (136.0, 143.0)	140.0 (135.0, 144.0)	139.5 (135.0, 145.0)	140.0 (136.0, 145.0)	0.5 (-2.0, 5.0)	0.0 (-3.0, 4.0)
	Wilcoxon signed rank p-value					.040	.11
	FDR q-value					.26	.38
Total Protein (g/dL)	n	38	38	38	38	38	38
	IQR Limits	7.1, 7.7	7.3, 7.8	6.9, 7.6	7.2, 7.6	-0.4, 0.1	-0.4, 0.0
	Mean (SD)	7.4 (0.5)	7.5 (0.5)	7.2 (0.6)	7.4 (0.5)	-0.2 (0.4)	-0.1 (0.4)
	Median (Range)	7.5 (5.9, 8.3)	7.5 (6.5, 8.4)	7.3 (5.8, 8.2)	7.4 (5.6, 8.2)	-0.1 (-1.8, 0.4)	-0.1 (-0.9, 1.1)
	Wilcoxon signed rank p-value					.012	.009
	FDR q-value					.11	.098

Abbreviations: FDR, false discovery rate; IQR, interquartile range, n, sample size; SD, standard deviation

Footnote:

The within group paired changed from baseline (day −7) to day 42 was compared with the Wilcoxon sign rank test. A false discovery rate (FDR; q-value) adjustment was used to control for multiple testing. If the within group paired change was significant ($q < .05$), then the within subject change in safety blood chemistry and hematology was compared between products with the Wilcoxon rank sum test.

Supplemental Table 5. Fasting hematology

Test	Statistic	Day −7		Day 42		Change	
		**BS50**	**Placebo**	**BS50**	**Placebo**	**BS50**	**Placebo**
Basophil (%)	n	38	38	38	38	38	38
	IQR Limits	0.6, 1.0	0.5, 0.9	0.6, 1.1	0.6, 1.1	-0.1, 0.3	-0.1, 0.2
	Mean (SD)	0.8 (0.4)	0.8 (0.3)	0.9 (0.4)	0.8 (0.3)	0.1 (0.3)	0.1 (0.2)
	Median (Range)	0.8 (0.3, 2.3)	0.8 (0.1, 1.4)	0.8 (0.3, 2.0)	0.8 (0.3, 1.3)	0.1 (-0.5, 0.7)	0.1 (-0.4, 0.7)
	Wilcoxon signed rank p-value					.053	.24
	FDR q-value					.31	.65
Basophil Absolute (K/UL)	n	38	38	38	38	38	38
	IQR Limits	0.0, 0.1	0.0, 0.1	0.0, 0.1	0.0, 0.1	-0.0, 0.0	-0.0, 0.0
	Mean (SD)	0.1 (0.0)	0.0 (0.0)	0.1 (0.0)	0.0 (0.0)	0.0 (0.0)	0.0 (0.0)
	Median (Range)	0.1 (0.0, 0.1)	0.1 (0.0, 0.1)	0.1 (0.0, 0.1)	0.1 (0.0, 0.1)	0.0 (-0.0, 0.0)	0.0 (-0.0, 0.0)
	Wilcoxon signed rank p-value					.17	.45
	FDR q-value					.50	.78
Eosinophil (%)	n	38	38	38	38	38	38
	IQR Limits	1.9, 4.3	1.7, 3.6	2.2, 5.2	1.7, 3.5	-0.4, 1.1	-0.4, 0.7
	Mean (SD)	3.5 (2.4)	2.9 (1.9)	3.8 (2.3)	3.1 (2.0)	0.4 (1.9)	0.2 (1.1)
	Median (Range)	2.7 (0.6, 12.1)	2.6 (0.5, 8.8)	3.4 (0.8, 11.5)	2.7 (0.9, 11.2)	0.3 (-6.9, 5.5)	0.2 (-2.9, 2.4)
	Wilcoxon signed rank p-value					.077	.36
	FDR q-value					.33	.75

Test	Statistic	Day −7		Day 42		Change	
		BS50	Placebo	BS50	Placebo	BS50	Placebo
Eosinophil Absolute (K/UL)	n	38	38	38	38	38	38
	IQR Limits	0.1, 0.3	0.1, 0.2	0.1, 0.3	0.1, 0.2	-0.0, 0.1	-0.0, 0.0
	Mean (SD)	0.2 (0.1)	0.2 (0.1)	0.2 (0.1)	0.2 (0.1)	0.0 (0.1)	0.0 (0.1)
	Median (Range)	0.2 (0.0, 0.6)	0.2 (0.0, 0.7)	0.2 (0.0, 0.7)	0.2 (0.1, 0.6)	0.0 (-0.2, 0.3)	0.0 (-0.1, 0.1)
	Wilcoxon signed rank p-value					.085	.40
	FDR q-value					.33	.78
Hematocrit (%)	n	38	38	38	38	38	38
	IQR Limits	40.3, 44.6	39.7, 45.5	39.7, 44.0	39.5, 44.3	-1.4, 1.2	-1.5, 1.0
	Mean (SD)	42.7 (3.5)	42.6 (3.6)	42.3 (3.6)	42.2 (3.4)	-0.4 (2.0)	-0.4 (1.8)
	Median (Range)	42.2 (36.7, 50.0)	42.3 (37.8, 53.5)	41.5 (37.0, 52.6)	41.9 (35.3, 52.3)	-0.5 (-5.5, 3.1)	0.1 (-4.7, 2.1)
	Wilcoxon signed rank p-value					.35	.44
	FDR q-value					.75	.78
Hemoglobin (g/dL)	n	38	38	38	38	38	38
	IQR Limits	13.3, 14.8	12.8, 15.0	13.2, 15.0	13.0, 14.8	-0.4, 0.3	-0.3, 0.3
	Mean (SD)	14.1 (1.2)	13.9 (1.4)	14.0 (1.3)	13.9 (1.3)	-0.1 (0.6)	-0.1 (0.5)
	Median (Range)	13.9 (11.9, 16.8)	13.8 (11.7, 17.4)	13.8 (11.8, 17.6)	13.6 (12.0, 16.9)	-0.0 (-1.9, 1.1)	0.1 (-1.2, 0.7)
	Wilcoxon signed rank p-value					.61	.95
	FDR q-value					.78	.96

Test	Statistic	Day −7		Day 42		Change	
		BS50	Placebo	BS50	Placebo	BS50	Placebo
Lymphocyte (%)	n	38	38	38	38	38	38
	IQR Limits	29.0, 35.9	29.4, 37.2	25.6, 35.1	29.8, 37.8	-3.7, 2.4	-2.4, 3.0
	Mean (SD)	32.1 (6.2)	33.4 (6.2)	30.9 (8.1)	33.5 (6.9)	-1.2 (5.7)	0.1 (7.1)
	Median (Range)	33.6 (14.2, 44.6)	32.5 (22.7, 50.1)	31.5 (15.5, 51.3)	32.8 (19.5, 52.4)	-1.2 (-16.1, 15.5)	0.4 (-13.1, 20.8)
	Wilcoxon signed rank p-value					.27	.97
	FDR q-value					.65	.97
Lymphocyte Absolute (K/UL)	n	38	38	38	38	38	38
	IQR Limits	1.5, 2.5	1.7, 2.2	1.4, 2.1	1.7, 2.2	-0.3, 0.1	-0.2, 0.1
	Mean (SD)	2.0 (0.6)	2.1 (0.5)	1.9 (0.6)	2.0 (0.5)	-0.1 (0.4)	-0.0 (0.3)
	Median (Range)	1.8 (1.1, 3.6)	2.1 (1.2, 3.2)	1.8 (0.9, 3.3)	1.9 (1.2, 3.2)	-0.1 (-1.0, 0.7)	-0.0 (-0.8, 0.7)
	Wilcoxon signed rank p-value					.030	.46
	FDR q-value					.21	.78
Mean Corpuscular Hemoglobin (pg)	n	38	38	38	38	38	38
	IQR Limits	29.4, 31.7	29.3, 30.9	29.8, 31.5	28.9, 31.0	-0.2, 0.6	-0.3, 0.2
	Mean (SD)	30.5 (1.4)	29.6 (2.3)	30.7 (1.3)	29.6 (2.4)	0.1 (0.5)	-0.0 (0.4)
	Median (Range)	30.5 (27.0, 33.4)	30.0 (20.4, 34.1)	30.7 (27.7, 33.2)	30.0 (20.0, 34.2)	0.0 (-0.8, 1.3)	0.0 (-0.7, 0.8)

Test	Statistic	Day −7		Day 42		Change	
		BS50	Placebo	BS50	Placebo	BS50	Placebo
	Wilcoxon signed rank p-value					.17	.94
	FDR q-value					.50	.96
Mean Corpuscular Hemoglobin Concentration (g/dL)	n	38	38	38	38	38	38
	IQR Limits	32.4, 33.5	32.0, 33.2	32.7, 33.6	32.1, 33.7	-0.2, 0.8	-0.1, 0.6
	Mean (SD)	33.0 (0.8)	32.6 (1.0)	33.2 (0.9)	32.8 (1.1)	0.2 (0.7)	0.2 (0.5)
	Median (Range)	32.8 (31.8, 34.9)	32.8 (29.9, 34.7)	33.0 (31.4, 35.1)	32.9 (30.7, 35.1)	0.2 (-1.5, 1.6)	0.2 (-0.9, 1.0)
	Wilcoxon signed rank p-value					.059	.026
	FDR q-value					.31	.21
Mean Corpuscular Volume (fL)	n	38	38	38	38	38	38
	IQR Limits	89.5, 95.6	89.0, 94.0	89.3, 95.2	89.1, 93.4	-1.0, 0.7	-1.3, 0.8
	Mean (SD)	92.5 (4.1)	90.8 (5.9)	92.4 (3.7)	90.3 (6.1)	-0.1 (1.7)	-0.5 (1.5)
	Median (Range)	92.4 (82.1, 100.3)	91.9 (65.2, 103.0)	92.0 (84.5, 99.3)	91.1 (64.0, 101.1)	-0.4 (-3.0, 6.3)	-0.7 (-5.1, 2.6)
	Wilcoxon signed rank p-value					.28	.069
	FDR q-value					.65	.32
Monocyte (%)	n	38	38	38	38	38	38
	IQR Limits	7.4, 10.2	7.7, 10.2	7.6, 9.9	8.0, 9.6	-0.9, 0.9	-0.6, 0.7

Test	Statistic	Day −7		Day 42		Change	
		BS50	Placebo	BS50	Placebo	BS50	Placebo
	Mean (SD)	8.8 (1.9)	9.0 (1.8)	8.7 (1.4)	8.9 (1.5)	-0.1 (1.2)	-0.1 (1.4)
	Median (Range)	8.5 (5.3, 13.1)	8.7 (5.8, 12.8)	8.6 (6.0, 11.0)	8.8 (6.0, 13.9)	-0.3 (-2.6, 3.0)	-0.1 (-3.6, 2.6)
	Wilcoxon signed rank p-value					.55	.85
	FDR q-value					.78	.94
Monocyte Absolute (K/UL)	n	38	38	38	38	38	38
	IQR Limits	0.4, 0.6	0.5, 0.6	0.4, 0.6	0.4, 0.6	-0.1, 0.1	-0.1, 0.0
	Mean (SD)	0.5 (0.1)	0.6 (0.1)	0.5 (0.1)	0.5 (0.2)	-0.0 (0.1)	-0.0 (0.1)
	Median (Range)	0.5 (0.3, 1.0)	0.5 (0.3, 1.0)	0.5 (0.3, 0.8)	0.5 (0.3, 1.1)	-0.0 (-0.3, 0.2)	-0.0 (-0.3, 0.4)
	Wilcoxon signed rank p-value					.60	.43
	FDR q-value					.78	.78
Neutrophil (%)	n	38	38	38	38	38	38
	IQR Limits	50.5, 58.7	50.3, 57.9	49.8, 62.1	47.7, 58.1	-4.2, 4.9	-3.0, 3.7
	Mean (SD)	54.5 (6.6)	53.6 (7.1)	55.2 (8.6)	53.4 (7.1)	0.7 (7.5)	-0.3 (8.0)
	Median (Range)	53.3 (42.7, 72.6)	54.5 (34.4, 69.8)	55.2 (38.6, 71.7)	54.3 (38.2, 70.0)	-0.3 (-18.5, 25.1)	0.6 (-22.8, 16.7)
	Wilcoxon signed rank p-value					.74	.87
	FDR q-value					.85	.94

Test	Statistic	Day −7		Day 42		Change	
		BS50	Placebo	BS50	Placebo	BS50	Placebo
Neutrophil Absolute (K/UL)	n	38	38	38	38	38	38
	IQR Limits	2.6, 4.1	2.8, 3.9	2.7, 4.0	2.7, 3.9	-0.5, 0.5	-0.5, 0.4
	Mean (SD)	3.4 (1.1)	3.4 (1.1)	3.5 (1.2)	3.3 (1.0)	0.0 (1.0)	-0.1 (1.2)
	Median (Range)	3.4 (1.8, 7.3)	3.3 (1.4, 6.1)	3.2 (1.7, 7.5)	3.3 (1.4, 5.9)	-0.1 (-2.7, 2.7)	-0.1 (-3.8, 2.2)
	Wilcoxon signed rank p-value					.62	.57
	FDR q-value					.78	.78
Platelet (K/UL)	n	38	38	38	38	38	38
	IQR Limits	222.0, 291.0	211.0, 265.0	229.0, 308.0	215.0, 272.0	-7.0, 14.0	-13.0, 25.0
	Mean (SD)	258.2 (54.3)	241.8 (41.0)	266.4 (60.6)	245.2 (44.2)	8.2 (24.3)	3.4 (25.1)
	Median (Range)	245.5 (168.0, 396.0)	238.0 (159.0, 340.0)	256.5 (144.0, 435.0)	231.5 (187.0, 370.0)	4.0 (-34.0, 75.0)	0.0 (-55.0, 58.0)
	Wilcoxon signed rank p-value					.096	.67
	FDR q-value					.36	.80
Red Blood Cell Distribution Width (%)	n	38	38	38	38	38	38
	IQR Limits	12.2, 13.0	12.2, 13.1	11.9, 12.9	12.1, 12.9	-0.4, 0.0	-0.3, 0.2
	Mean (SD)	12.7 (0.8)	12.8 (1.2)	12.6 (0.8)	12.8 (1.2)	-0.2 (0.4)	-0.0 (0.3)
	Median (Range)	12.6 (11.1, 15.8)	12.7 (11.2, 18.0)	12.5 (11.6, 15.4)	12.7 (11.2, 18.1)	-0.2 (-1.2, 0.7)	-0.1 (-0.7, 0.6)

Test	Statistic	Day −7		Day 42		Change	
		BS50	Placebo	BS50	Placebo	BS50	Placebo
	Wilcoxon signed rank p-value					.008	.37
	FDR q-value					.098	.76
Red Blood Cell Distribution Width Standard Deviation	n	38	38	38	38	38	38
	IQR Limits	41.6, 45.6	40.4, 44.4	40.8, 44.1	40.1, 44.1	-1.7, 0.4	-1.3, 0.4
	Mean (SD)	43.3 (2.9)	42.3 (2.7)	42.7 (3.0)	41.9 (2.6)	-0.6 (1.8)	-0.4 (1.4)
	Median (Range)	43.4 (37.8, 51.9)	42.3 (36.1, 48.2)	42.4 (38.1, 50.8)	41.6 (36.1, 47.3)	-0.6 (-5.1, 4.4)	-0.3 (-3.6, 2.4)
	Wilcoxon signed rank p-value					.018	.081
	FDR q-value					.16	.33
Red Blood Cell (M/UL)	n	38	38	38	38	38	38
	IQR Limits	4.3, 4.9	4.3, 5.1	4.2, 4.9	4.4, 5.0	-0.1, 0.1	-0.1, 0.1
	Mean (SD)	4.6 (0.5)	4.7 (0.6)	4.6 (0.5)	4.7 (0.6)	-0.0 (0.2)	-0.0 (0.2)
	Median (Range)	4.5 (3.8, 6.1)	4.6 (3.7, 6.9)	4.4 (3.8, 6.0)	4.6 (3.7, 6.9)	-0.1 (-0.6, 0.5)	0.0 (-0.5, 0.3)
	Wilcoxon signed rank p-value					.28	.80
	FDR q-value					.65	.90
White Blood Cell (K/UL)	n	38	38	38	38	38	38
	IQR Limits	5.0, 7.0	5.3, 6.9	5.2, 7.2	5.3, 6.7	-0.7, 0.6	-0.9, 0.6

Test	Statistic	Day −7		Day 42		Change	
		BS50	Placebo	BS50	Placebo	BS50	Placebo
	Mean (SD)	6.3 (1.5)	6.3 (1.4)	6.2 (1.6)	6.1 (1.4)	-0.1 (1.2)	-0.2 (1.3)
	Median (Range)	6.5 (3.7, 10.0)	6.1 (3.9, 9.6)	5.9 (3.5, 10.5)	6.1 (3.2, 10.2)	-0.1 (-3.2, 2.3)	-0.1 (-3.9, 2.2)
	Wilcoxon signed rank p-value					.53	.48
	FDR q-value					.78	.78

Abbreviations: FDR, false discovery rate; IQR, interquartile range, n, sample size; SD, standard deviation

Footnote:

The within group paired changed from baseline (day −7) to day 42 was compared with the Wilcoxon sign rank test. A false discovery rate (FDR; q-value) adjustment was used to control for multiple testing. If the within group paired change was significant ($q < .05$), then the within subject change was compared between products with the Wilcoxon rank sum test.

Supplemental Table 6. *In vitro* secreted enzymatic activity of *B. subtilis* BS50

Enzyme category	Activity
Amylase	++
Cellulase	+++
Lipase	++
Protease	+++

Footnote: Agar-based screens were used to determine the relative amounts of enzymes produced by BS50. For protease activity, skim milk (SM) agar plates were prepared per manufacturer instructions (product no. M763, HiMedia Laboratories, LLC; Kennett Square, PA, USA), inoculated with 15 µL of BS50 standardized culture (0.5 McFarland standard) or 15 µL of a known concentration of protease solution (positive control), and subsequently incubated at 35°C for 18–24 hours. For amylase activity, starch agar plates were prepared per manufacturer instructions (product no. M107S; HiMedia Laboratories, LLC), inoculated with 15 µL of BS50 standardized culture (0.5 McFarland standard) or 15 µL of a known concentration of amylase solution (positive control), and subsequently incubated at 35°C for 18–24 hours. After incubation, plates were stained with Gram's iodine (50% solution) for 15 minutes. Lipolytic activity was evaluated using 1% tributyrin phenol red (TPR) agar plates.[1] TPR agar plates were prepared, inoculated with 15 µL of BS50 standardized culture (0.5 McFarland standard) or 15 µL of a known concentration of lipase solution (positive control), and subsequently incubated at 35°C for 18–24 hours. Cellulase activity was evaluated using carboxymethylcellulose (CMC) agar.[2] CMC agar plates were prepared, inoculated with 15 µL of BS50 standardized culture (0.5 McFarland standard) or 15 µL of a known concentration of cellulase solution (positive control), and subsequently incubated at 35°C for 18–24 hours. To visualize clearing zones, the agar surface was flooded with 0.1% Congo red for 15 minutes followed by 1M NaCl solution rinse. The clearing zones (i.e., enzyme activity) were measured in millimeters from the edge of bacterial growth to the edge of clearing. The number of plus signs indicates the relative size of the clearing zones, and thus, the relative amount of enzymatic activity. (+): clearing zones < 5 mm; (++): clearing zones > 5 mm and < 10 mm; (+++): clearing zones > 15 mm.

References:

1. Ramnath L, Sithole B, Govinden R. Identification of lipolytic enzymes isolated from bacteria indigenous to eucalyptus wood species for application in the pulping industry. Biotechnol Rep (Amst). 2017;15:114–124. doi:10.1016/j.btre.2017.07.004.
2. Ponnambalam AS, Deepthi RS, Ghosh AR. Qualitative display and measurement of enzyme activity of isolated cellulolytic bacteria. Biotechnol Bioinf Bioeng. 2011;1(1):33–37.

Supplemental Table 7. *In vitro* antimicrobial activity of *B. subtilis* BS50

Gram reaction and strains	Antimicrobial activity
Gram-positive bacteria	
Listeria monocytogenes ATCC 19114	++
Staphylococcus aureus subsp. *aureus* ATCC 25923	+
Streptococcus agalactiae ATCC 12403	+++
Gram-negative bacteria	
Bordetella bronchiseptica NRRL B-140	++
Escherichia coli O157:H7 ATCC 43888	+
Pasteurella multocida ATCC 12945	nz
Pseudomonas aeruginosa ATCC 27853	nz
Salmonella enterica subsp. *enterica* Abaetetuba ATCC 35640	nz
Salmonella heidelberg ATCC 8326	+
Salmonella typhimurim ATCC 13311	nz

Abbreviations: nz, no zone of inhibition

Footnote: The antimicrobial activity of BS50 was determined by a cross-streak method. Trypticase soy agar (TSA) plates were prepared, inoculated with BS50 by a single streak of pure culture down the center of the Petri plate, and incubated at 35°C for 18–24 hours. The plates were then seeded with various pathogenic bacterial strains by a single streak perpendicular to the BS50 center streak. Plates were further incubated at 35°C for 18–24 hours. Antimicrobial activity was measured as the distance (mm) between the BS50 center streak and the growth of the perpendicular streak. The number of plus signs indicates the relative size of the clearing zones, and thus, the relative amount of antimicrobial activity. (+): clearing zones < 5 mm; (++): clearing zones > 5 mm and < 10 mm; (+++): clearing zones > 15 mm. Each experiment was repeated at least three times, and the mean values were used for inhibition zone scoring.
